# A Context-Adapted Diabetes Prevention Program (Small Steps for Big Changes) in Australia: Protocol for a Hybrid Implementation-Effectiveness Study

**DOI:** 10.2196/81195

**Published:** 2026-01-19

**Authors:** Genevieve N Healy, Sjaan R Gomersall, David W Dunstan, Elisabeth A H Winkler, Katherine A Heseltine, Grace Q Sim, Fiona Mason, Denis Giguere, Michael Tsiamis, Mary E Jung, Ana D Goode

**Affiliations:** 1 Health and Wellbeing Centre for Research Innovation School of Human Movement and Nutrition Sciences The University of Queensland Brisbane, Queensland Australia; 2 School of Health and Rehabilitation Sciences The University of Queensland Brisbane Australia; 3 Institute for Physical Activity and Nutrition, School of Exercise and Nutrition Sciences Deakin University Burwood, BC Australia; 4 Physical Activity Laboratory Baker Heart and Diabetes Institute Melbourne, Victoria Australia; 5 School of Human Movement and Nutrition Sciences The University of Queensland Brisbane Australia; 6 Logan Healthy Living UQ Health Care Logan Australia; 7 The Y Queensland Brisbane Australia; 8 School of Health and Exercise Sciences Faculty of Health and Social Development University of British Columbia Kelowna, BC Canada

**Keywords:** diabetes prevention, community, exercise, diet, motivational interviewing, cultural adaptation, implementation, intervention

## Abstract

**Background:**

With type 2 diabetes rates escalating worldwide, including in Australia, effective, acceptable, sustainable, and scalable diabetes prevention programs are needed. Small Steps for Big Changes (SSBC) is a Canadian-developed, community-delivered diet and exercise counseling intervention for individuals at risk of type 2 diabetes. The 3- to 6-week intervention can be delivered by trained non–health professionals, with all SSBC coaches receiving training in the delivery of the program, including motivational interviewing. However, the suitability and feasibility of the program in the Australian context are unknown. To address this gap, funding has been secured to adapt, implement, and evaluate SSBC in Australia (SSBC Australia), providing evidence on its effectiveness, acceptability, and implementation in this context.

**Objective:**

The aim of the study is to describe the protocol for the type 2 cluster nonrandomized single-arm hybrid effectiveness-implementation trial of SSBC Australia.

**Methods:**

SSBC Australia will be delivered and evaluated in 5 community-based sites across 2 organizations in South-East Queensland, Australia. One organization (1 site) will trial training students on clinical and project placements as coaches. The evaluation period is 4 years. For the first 2 years, sites receive funding for program delivery, after which, ongoing delivery will be self-funded. The recruitment target is 500 participants completing the 6-session intervention across the 5 sites within 2 years, with approximately 50 coaches trained. Data will be collected from the organization, site, coach, and client using a variety of methods (surveys, objective assessments, interviews, site audits, website analytics, meeting minutes, and project tracking). The integrated Practical, Robust, Implementation Sustainability Model and Reach, Effectiveness, Adoption, Implementation, Maintenance framework and the Affordability, Practicality, Effectiveness and Cost-Effectiveness, Acceptability, Side-Effects/Safety, and Equity criteria will guide implementation and evaluation and inform iterative adaptations as required. Data will be collected on the context for delivery; adoption and reach (number of coaches and clients and their characteristics); effectiveness of the coach training and the intervention (client pre- and post changes in measured clinical indicators [body composition, cardiorespiratory fitness, strength, and balance], self-reported health behaviors [movement behaviors, healthy eating, and program behaviors], psychosocial indicators [self-efficacy and social supports], quality of life, diabetes status, and health care use); implementation of the coach training and program delivery (fidelity and acceptability); and maintenance of program delivery (sites) and client outcomes at 3, 6, and 12 months post end-of-program.

**Results:**

Ethics approval and trial registration were completed. All 5 sites have been recruited and undergone preimplementation evaluation, with SSBC Australia coach training underway. Client recruitment started in September 2025.

**Conclusions:**

This study will provide evidence whether the contextually adapted SSBC diabetes prevention program can be successfully implemented and is effective within an Australian context. Findings will inform potential expansion to additional community sites and health service contexts.

**Trial Registration:**

Australian and New Zealand Clinical Trials Registry ACTRN12624001194550; https://tinyurl.com/588jkmva

**International Registered Report Identifier (IRRID):**

DERR1-10.2196/81195

## Introduction

Type 2 diabetes is a global epidemic: in 2021, an estimated 529 million adults had diabetes with projections that this will reach 1.31 billion by 2050 [1]. Given the significant health, social, and financial costs of diabetes (eg, diabetes costs an estimated US $2.25 billion to the Australian health care system in 2020-2021 [2]), there has been significant investment in understanding how to prevent and reduce the risk of developing this chronic condition. The pivotal US Diabetes Prevention Program demonstrated that a lifestyle modification intervention involving physical activity and dietary changes was effective at reducing progression to type 2 diabetes in those at elevated risk [3], with these effects sustained long-term [4]. Diabetes prevention programs have now been adapted for community-based delivery across multiple communities, with demonstrated cost-effectiveness [5], and effectiveness robust to various cultural adaptations and translational strategies [6]. However, the length and intensity of these programs, which typically require at least a 12-month commitment, impact on their reach, retention, and maintenance [7,8]. Lower intensity programs (ie, shorter duration and fewer sessions) may be more attractive to potential participants and align with calls to action to reconsider how diabetes prevention is approached [9]; however, the evidence for these programs is currently relatively limited [10]. One such lower-intensity diabetes prevention program is Small Steps for Big Changes (SSBC).

SSBC is an evidence- and community-based diet and exercise counseling intervention for individuals at risk of type 2 diabetes [11-15]. The program, developed by Jung et al [15] in Canada, was designed for feasible and sustainable translation into communities nationally [11,16] and enhanced to assist in providing equitable access and inclusive care [14], with the development, efficacy testing, and scale-up of the SSBC program within the Canadian context described in detail elsewhere [11,12,14,16,17]. The intervention was based on social cognitive theory [18] and behavior change techniques specifically known for enhancing adherence to diet and exercise modification [13]. Mapping to the behavior change taxonomy identified 43 behavior change techniques used as part of the SSBC program [12]. The implementation strategies of SSBC have been iteratively tested as part of the research-to-practice and scale-up process.

A key implementation strategy tested was the use of lay health coaches to deliver the program. Here, coaches attended a 3-day in-person workshop where they were trained in motivational interviewing (MI) as well as the diet, exercise, and diabetes content of the program. This strategy was successfully tested with a community-based partner (YMCA) [16,19-21]. To further enhance scalability, this training was adapted to be delivered online using an integrated knowledge translation approach [22] and shown to be successful and acceptable to coaches [11,21]. The coaches deliver the 6-session program within community fitness facilities (eg, YMCAs). Each session lasts approximately 60-75 minutes and is comprised of approximately 40 minutes of MI-informed one-on-one counseling on diet and exercise behavior and 20-30 minutes of structured exercise at either a moderate or high intensity. The ability for community members and previous participants to take on the role as coach distinguishes SSBC from other diabetes prevention programs that typically require delivery by health professionals. Findings to date have demonstrated that this delivery approach is feasible, acceptable, and effective [11,16,23]. Based on the success of these findings, funding was granted to Jung et al [24] to expand the delivery of the SSBC program in YMCA sites across Canada, including in areas with low socioeconomic status and ethnoculturally diverse populations. Details on the implementation strategies used in the promotion, implementation, and evaluation of the Canadian delivery of the SSBC program, and the associated measurement of these strategies, will be reported in detail elsewhere [25].

Funding support was also granted to Healy et al [26] to understand the suitability, implementation, and effectiveness of the program within an Australian context. Like other countries, diabetes is a public health concern in Australia, with around 125 people a day diagnosed with type 2 diabetes and almost 1.2 million people living with the condition [2]. These rates vary by socioeconomic status, with age-standardized rates of type 2 diabetes in Australia almost twice as high in those living in the lowest socioeconomic areas (5.1%) compared to the highest socioeconomic areas (2.6%) [2]. Importantly, 1 in 6 Australian people have prediabetes: a condition in which blood glucose levels are higher than normal but not high enough to be classified as diabetes [27]. Notably, despite the position statement highlighting the need for screening of prediabetes [28], Australia does not yet have a national screening program for diabetes, so rates—particularly of prediabetes—may be an underestimate.

Australia offers a range of free diabetes prevention programs [29], with most provided at a state level and funded through government and Diabetes Australia or state-based partners. Various delivery modes are used, including telephone, online, and in-person, with most programs delivered by expert health professionals. In the state of Queensland, where SSBC in Australia (SSBC Australia) will be delivered, the freely available programs are My Health for Life [30] and Beat It [31]. My Health for Life is a government-funded 18-week program delivered over 6 sessions by a qualified health professional [30], while Beat It is a free 8-week program delivered by exercise professionals [31]. The Canadian-developed SSBC may provide an important addition to these offerings by providing an in-person program that does not require a health professional for delivery, extending the potential reach of the program into communities where such services are not readily available or where traditional diabetes prevention programs may not be widely accepted. The ability for SSBC to be delivered by a non–health professional may also increase the sustainability of the program.

Similar to Canada, the Australian implementation of SSBC (SSBC Australia) will have a delivery arm through the Queensland YMCA community sites (rebranded to The Y Queensland), with sites identified based on need and demand for such services [32], as well as providing a mix of regional and urban settings. Understanding the potential for delivery of SSBC Australia in regional settings is particularly important, given the geographical size of Queensland (1.727 million square kilometers, larger than all but 16 countries) and the population spread (more than half of the population lives outside the capital city area) [33] can make access to health care professionals to deliver diabetes prevention programs difficult. A community health care service, Logan Healthy Living by UQ Health Care, will also be included as a delivery site. UQ Health Care is a not-for-profit controlled entity of the University of Queensland. Logan Healthy Living is an interprofessional clinic with a range of allied health services that specializes in providing evidence-based interventions to manage and improve outcomes for individuals with various chronic diseases including those with and at risk of type 2 diabetes [34]. It uses a student-infused work-integrated learning placement model, with students from across multiple allied health professions and from both undergraduate and postgraduate programs working together to provide an interdisciplinary model of care [35]. One novel element of the SSBC Australia delivery will be trialing these placement students as SSBC coaches. In addition to providing a potentially sustainable delivery model, this also provides a mechanism to build the knowledge, skills, and confidence of these preprofessional students and enhance workforce capacity in lifestyle management interventions.

The Australian delivery of the SSBC program has been informed by a program of exploratory preimplementation work. This included a needs assessment within the community, preliminary discussions with the sites, and exploration of the appropriateness of the Canadian-developed SSBC coach training for the Australian context. The needs assessment was conducted via a cross-sectional survey with 575 community members of The Y (formerly known as The YMCA) across 20 Y Queensland sites [32]. Findings showed that there was both a need and a desire for a diabetes prevention program, with 46% of participants showing elevated risk of developing type 2 diabetes in the next 5 years according to the Australian Type 2 Diabetes Risk Assessment Tool (AUSDRISK) score [36], and 68% of participants expressing interest in taking part in such a program [32].

The preliminary site-level discussions involved interviews with site leads and other key staff members at each of the sites, with the Consolidated Framework for Implementation Research (CFIR) [37] used to guide the semistructured interview questions. An initial interview was conducted online, followed up with interviews on-site at each of the 5 sites. Findings highlighted that there was variation across sites in terms of the equipment available (both for assessment and exercise), services offered, staffing availability, and experience in implementing research projects and working with university partners. All sites catered to an aging population (appropriate for the SSBC intervention), were focused on serving the communities in which they worked, and had established communication channels available for program promotion. Findings confirmed that the sites identified for delivery were suitable, but site-specific protocols for implementation would be required.

The appropriateness of the online SSBC coach training for the Australian context was also explored, with findings reported in detail elsewhere [38]. In brief, focus groups and interviews, along with “think-alouds” of the online training experience, were conducted with a range of potential future coaches of SSBC Australia. These included current site staff, tertiary students, and allied health professionals and students who identify as Aboriginal or Torres Strait Islander. Overall, the SSBC program and the online coach training were well received and were seen as appropriate and feasible by all stakeholders. Key content modification suggestions were to reference the Australian physical activity and dietary guidelines (rather than the Canadian guidelines) and enhance the cultural safety components to be suitable for the Australian context. Key delivery modification suggestions were to allow strength training (which is now available as an augmented addition in the Canadian delivery) and provide opportunities to practice in a face-to-face setting prior to the mock session. These modifications were developed and tested with potential end users [39] and then incorporated into the coach training process and material for Australian delivery. The aim of this paper is to describe the protocol for the implementation and evaluation of SSBC Australia.

## Methods

### Study Design

In line with the SSBC Canada evaluation, SSBC Australia will be assessed via a type 2 hybrid effectiveness-implementation cluster nonrandomized single-arm trial, where the primary effectiveness outcome will be end-of-program change in self-reported moderate- to vigorous-intensity physical activity (MVPA) behavior, and the primary implementation outcome will be fidelity of program delivery within the provider settings. The study design with respect to establishing effectiveness is a cluster single-arm pre- and posttrial. The evaluation period is 4 years; however, research funding for site-level implementation is provided for the first 2 years only. The evaluation is facilitated by the research team across the 4 years. The timeline for adaption, delivery, and evaluation is shown in Table 1.

**Table 1:** Timeline of the project.

	Year 1	Year 2	Year 3	Year 4	Year 5
Site-level adaptations	✓				
Research-funded delivery		✓	✓		
Site-funded delivery				✓	✓
Research evaluation		✓	✓	✓	✓

### Frameworks for Evaluation and Reporting

The integrated Practical, Robust, Implementation Sustainability Model (PRISM) and Reach, Effectiveness, Adoption, Implementation, Maintenance (RE-AIM) framework [40] and the Affordability, Practicality, Effectiveness and Cost-Effectiveness, Acceptability, Side-Effects/Safety, and Equity (APEASE) criteria [41] will be used to guide the implementation and evaluation, assess progress over time, and make modifications as required. The PRISM and RE-AIM frameworks incorporate evaluation of contextual factors, implementation strategies, and RE-AIM outcomes. It has been widely used to understand the implementation of evidence-based programs into practice, including diabetes prevention programs [42]. Learnings from the Canadian implementation [25,43] as well as unique barriers and enablers within the Australian context will inform the SSBC Australia implementation strategies. In parallel and in collaboration with the Canadian SSBC research project [25], the SSBC Australia project will use the Expert Recommendations for Implementing Change taxonomy [44] and Proctor’s recommendations for specifying and reporting [45] to map, name, and define the implementation strategies for SSBC Australia, with this information to be reported in detail elsewhere. The Framework for Reporting Adaptations and Modifications to Evidence-Based Implementation Strategies [46] will be used to track adaptations to the implementation strategies. The SPIRIT (Standard Protocol Items: Recommendations for Interventional Trials) guidelines for reporting outcomes in trial protocols [47] and the StaRI (Standards for Reporting Implementation Studies) checklist [48] are used to guide reporting (Multimedia Appendix 1).

### Ethical Considerations

The study has received ethics approval from The University of Queensland Human Research Committee for the adaptation to the Australian context (#HE001455, #HE000500, and #HE001547), understanding of context and barriers and enablers to implementation from a site and provider perspective (#HE001455), and program delivery and evaluation (#HE001123 and #HE001280). All participants are provided with an electronic participant information and consent sheet prior to participation, where it is emphasized that participation in the research is voluntary. Participants can withdraw at any time without consequence; however, data collected up to that point may be used in analyses. Data are identifiable when collected and deidentified for analyses. Client participants are provided with up to 6 weeks of membership at their SSBC Australia site (with this paid for by the delivery site) and provided the program for free. Sites are provided with funding from the grant to deliver the program for the first 2 years.

### Aims of the SSBC Australia Trial

The overall aim of the trial is to evaluate the contextually adapted version of the Canadian-developed SSBC program (SSBC Australia). Specifically, we aim to evaluate the uptake (adoption) by coaches; reach of the program; program implementation, including program acceptability (at the provider site, coach, and client level) and contextual factors influencing implementation; program effectiveness; and sustainability of the program, including delivery costs and ongoing delivery.

### SSBC Australia Components

Implementation of the Canadian-developed SSBC program within the Australian context will involve four key elements: (1) understanding site-level requirements (site adaptations), (2) SSBC Australia coach training, (3) delivery of the program by site (delivery funded by research), and (4) delivery of the program by site (delivery funded by site).

#### Site Adaptations

In recognition that resource availability, workflows, and processes may differ between sites, understanding site adaptation requirements is built into the preimplementation phase process, with these processes designed and developed by SSBC Canada. A core part of this process is a preimplementation workshop. This workshop is to understand what site-level adaptations are needed as well as to clarify roles and responsibilities. This workshop is intended to build and extend on preliminary conversations with each site. Prior to the workshop, site leads will be sent a preworkshop checklist on elements to reflect on or ascertain prior to the workshop to help facilitate decision-making. The workshop will be recorded, and field notes taken and summarized. This summary will be shared with the site team and modified as required to appropriately reflect the discussions and decisions, both during the workshop and through other correspondence. Together, this information will be used to develop a site-level standard operating protocol and inform site-level implementation strategies.

#### SSBC Australia Coach Training

##### Overview

The SSBC Australia coach training consists of 4 main elements: the Canadian training modules, the Australian-specific modules and resources, the measurement assessment and exercise supervision training, and the competency evaluation. The Canadian and Australian modules are housed on an online training platform from the 3C Institute (Durham, North Carolina). A separate user journey is available for the Australian coaches so that they only access resources appropriate for SSBC Australia. A REDCap (Research Electronic Data Capture) coach training database was also created specifically for SSBC Australia (described in the SSBC Australia REDCap Coach Training Database section). Discussions around the training process will be held as part of the site preimplementation workshop, with any adaptions or additions to the training protocol (eg, the use of peers to practice skills prior to competency testing) for coaches recorded. In addition to any site-level variations, variations at an individual coach level will also be recorded via REDCap and asked via interviews.

##### Online Canadian Modules

Coaches will complete the Canadian modules as the first part of their SSBC training process. The online training consists of 7 modules, including a welcome and overview of the program, an overview of MI, and how to use the program resources, with each module including embedded quizzes [22]. The Canadian online training modules take approximately 3 to 4 hours to complete.

##### Australian-Specific Modules

The SSBC Australia coaches will then be asked to review 4 Australian-specific modules as part of their training process as well as the SSBC Australia client workbook (Table 2). These modules were designed to address the changes required to ensure that the program was suitable for delivery within the Australian context as well as provide training for the additional evaluation components that will be used in the Australian trial. An optional module is also provided, which provides further information on MI to complement the resources provided in the Canadian online training. Collectively, the Australian modules are expected to take approximately 2 hours to complete. Coaches can access these modules at any point via the online resource hub. This mechanism also provides flexibility to add additional training content if identified as part of the implementation process. Any additions will be tracked by the project team. Each module includes an Acknowledgement of Country at the start of the module in recognition that the research is being conducted on Aboriginal and Torres Strait Islander lands.

**Table 2:** Australian-specific modules for the Small Steps for Big Changes (SSBC) Australia implementation.

Module	Core content
1. Introduction to SSBC Australia and diabetes prevention	Introduction to SSBC Australia program Preventing type 2 diabetes (including Australian statistics) Physical activity (including reference to the importance of considering the 24-hour day) Healthy eating (in line with Australian dietary guidelines) Supporting behavior change
2. Fostering a safe and inclusive environment	Cultural safety in practice (Australian statistics) Guiding principles for culturally safe practice (adapted from Australian materials) Essentials for communicating clearly during appointments Additional cultural awareness resources (Australian-specific)
3. SSBC Australia coach guide	Overview of SSBC Australia (including summary of differences between SSBC Australia and SSBC Canada training) SSBC Australia data collection process Physical measurements protocol for SSBC Australia including demonstration videos SSBC Australia session guides (each session has a summary infographic, MI^a^ guide, and key resources) FAQ^b^ for SSBC Australia Additional resources (Australian specific)
4. Using REDCap^c^ in SSBC Australia	Overview of REDCap (SSBC Australia–specific) Scheduling guide for SSBC Australia Session guide for SSBC Australia Troubleshooting guide and FAQ
5. SSBC Australia client workbook	A copy of the SSBC Australia workbook clients receive
6. MI (optional)	Summary of core MI components (SPIRIT^d^, OARS^e^, and Ask-Tell-Ask) Processes that guide MI conversations with examples Change talk with examples

^a^MI: motivational interviewing.

^b^FAQ: frequently asked question.

^c^REDCap: Research Electronic Data Capture.

^d^SPIRIT: Standard Protocol Items: Recommendations for Interventional Trials.

^e^OARS: Open-Ended Questions, Affirmations, Reflections, Summaries.

##### Assessment Training and Exercise Supervision Training

Site leads or other trained staff will provide training in the processes required for the in-person measurements, completing the fidelity checklists, and supervising the exercise sessions.

##### Competency Assessment

Coaches are required to undertake a competency assessment prior to delivery of the SSBC Australia program. The protocol and standards for coach competency assessment were designed and tested by SSBC Canada. In line with the Canadian model, assessment of competency includes 4 elements: knowledge of the program content, ability to conduct the in-person measurement protocol, ability to appropriately supervise the exercise sessions (or provide guidance on appropriate exercise sessions to attend if a site chooses to use group-based exercise), and ability to use appropriate MI skills. Knowledge will be assessed via a 20-item survey, which has been adapted from the Canadian knowledge check to align with the Australian-specific content. A pass mark of at least 70% (14/20) is required to achieve competency, with coaches able to repeat the survey as required until they meet this pass mark. Scores and the number of passes on the first attempt will be recorded. The site-level trainer or the research team will assess competency in the in-person measurement and exercise supervision protocol. For assessment of competency in MI, coaches will undertake a mock session of the first health coaching session, with assessment conducted by the research team using the Abbreviated Motivational Interviewing Competency Assessment tool [49] and a checklist against delivery of session content. In line with the Canadian SSBC coach training [11,22], the coach will be provided with feedback on their mock session by the research team (who have been trained in the use of the Abbreviated Motivational Interviewing Competency Assessment tool), with a minimum score of 0.5 of 2.0 for each of the 5 criteria required to meet competency. Potential coaches can repeat any component or part thereof to meet competence.

##### SSBC Australia REDCap Coach Training Database

An Australian-specific implementation strategy was the development of a REDCap database to allow staff to easily guide and track the coach training process and evaluate the training. The database also enabled the collection of data from coaches regarding their perspectives on the SSBC Australia program at various intervals after completing the training. For student coaches, data were collected at the end of their placement and at 1 month and 1 year later; for all other coaches, data were collected at 2 months, 1 year, 2 years, 3 years, and 4 years after the training. Research staff and site staff performing the training use this database to add records for new trainee coaches, send out the required training steps in order, receive alerts indicating when each step is done, fill in competency checklists for the mock session assessments (MI, measurement, and exercise supervision), and send iterative and final feedback to the trainees, as well as to notify relevant staff when competent coaches will require access to the REDCap client database. The steps trainees perform are completing consent forms, completing pretraining surveys, following instructions to join the Online 3C Training platform (and undertake that training), successfully complete a knowledge check or a supplemental knowledge check (if the first check is failed), and use links they are sent of the practice coaching forms for the mock session assessments. After they have completed their mock session assessments, they complete posttraining surveys, receive final feedback on their mock assessments, and complete ongoing surveys tracking training outcomes and their perspectives on the training at various time points after the training.

#### Delivery of Program by Site (Delivery Funded by Research)

For the first 2 years, the delivery of the SSBC Australia intervention to clients will be primarily funded by the research grant, with in-kind support given by the delivery provider in relation to free gymnasium membership for the duration of the 6-session delivery (4 to 6 weeks).

Aligned with the Canadian model, the SSBC Australia intervention involves 2 key elements: the 6 individual coaching and exercise sessions with the trained SSBC coach and the client workbook.

##### Individual Coaching Sessions

The 6 individual sessions are delivered using an MI approach across a 3- to 4-week period (noting additional time up to 6 weeks is allowable if required by the site or client). Each session involves a coaching component for approximately 30-40 minutes and an exercise component for 20-30 minutes. Sessions 1 and 6 also have an assessment component (see Clinical Indicators (Objective Physical Assessments) section). Each coaching session includes a check-in with the client, provision of information around a session topic, planning and goal setting, and wrap-up.

Session topics covered in SSBC Australia include an introduction to the SSBC Australia program; healthy eating and identifying hidden sugars; physical sensations of cardiovascular exercise and strength training; carbohydrates, glycemic index, and a healthy plate; identifying barriers and enablers to active living; and managing setbacks and planning for success. Key changes from the SSBC Canada program were the alignment of recommendations to the Australian Physical Activity [50] and Healthy Eating [51] guidelines. This included the addition of material on hidden sugars, strength training (in line with evidence of the benefits of strength training for diabetes prevention [52]), and the importance of considering the 24-hour day [53], including the identification and impact of high sedentary time [54]. Selected by the participant, each exercise session involves either moderate-intensity continuous training or high-intensity interval training, with the duration of the exercise component increasing across the 6 sessions from 12 to 20 minutes in the first session, up to 30 minutes in each of the last 2 sessions. Delivery sites can choose to substitute or augment this core program with tailored elements such as virtual delivery of the coaching and use of group-based exercise classes. Provision and take-up of any substitutions or augmentations will be tracked. Scripts embedded in REDCap will be used to guide SSBC Australia coaches through the key points to cover in each session, in conjunction with data collection to monitor implementation fidelity.

##### Client Workbook

Clients will be provided with an SSBC Australia workbook, which will be used to guide the session activities. In addition to the educational content and session-based activities, there is also space for clients to monitor and track their progress toward their goals and reflect on their progress if they choose. The SSBC workbook was adapted to be suitable for an Australian delivery context (eg, the use of Australian rather than Canadian guidelines and examples of Australian rather than Canadian foods).

#### Community of Practice

The SSBC Canada team developed 2 communities of practice: one for SSBC coaches and one for site leads implementing the program. The Australian coaches and site leads will be invited to these virtual communities of practice to enhance and encourage learnings across all implementation sites. Engagement with the Canadian community-of-practice site will be monitored through the Canadian website [55] and a Microsoft Teams site. In addition, an online sharing site (Microsoft Teams) will be established to facilitate an Australian-based community-of-practice among SSBC Australia coaches and site leads at participating Australian sites, with engagement and meeting minutes recorded and reviewed.

#### Delivery of Program by Site (Delivery Funded by Site)

After 2 years of primarily research-funded delivery, sites will have the opportunity to decide whether they want to continue to deliver the program (with sites fully funding the delivery) or to not continue delivery. The research grant will continue to fund the evaluation of the program until the end of 4 years of implementation regardless of whether sites choose to continue the program or not after 2 years. Site and organization leads have committed to this evaluation for 4 years. A sustainability adaptation workshop will be conducted with each site that decides to continue delivery at both the end of year 2 (end of research-funded delivery) and the end of year 4 (end of research evaluation). SSBC Australia will be considered affordable on the APEASE criteria if the site decides to continue delivery of the program after 2 years.

### SSBC Australia Participants: Delivery Providers

#### Overview

SSBC Australia will be delivered across 2 providers and 5 sites in South-East Queensland: The Y Queensland (4 sites) and Logan Healthy Living (1 site). These sites were identified (1) to align with the delivery partner in Canada (Y Queensland) and (2) to address an identified need for a diabetes prevention program, which can be delivered by non–health professionals (including students), from a community-based primary care partner (Logan Healthy Living). Identification and recruitment of sites occurred as part of the grant funding submission process.

#### The Y Queensland

The Y Queensland is a not-for-profit organization with services including childcare, fitness, camping, education, recreation, and community programs [56]. The organization is aligned with the YMCA’s in Canada, which are the delivery sites for the SSBC Canada program [16]. There are 9 Y sites across South-East Queensland. In partnership with The Y leadership team, 4 sites were chosen as delivery sites for SSBC Australia: 1 inner city, 1 outer city, and 2 regional. It was expected that these sites would provide an appropriate mix of participants and resource availability to be able to inform the sustainability and potential scale-up of the program.

#### Logan Healthy Living

Logan Healthy Living by UQ Health Care is a community-based allied health service designed to support adults living in the Logan area with, or at risk of, type 2 diabetes [34]. Logan is a culturally diverse and relatively low-income region in South-East Queensland. The region experiences rates of diabetes higher than the Queensland average (5.2% vs 4.5% [57]), with diabetes the most common cause of potentially preventable hospitalizations [58]. Logan Healthy Living was designed to address this need, with details of the clinic development and service provision reported previously [34]. In brief, this multisectorial health hub model, which was established in 2021, is delivered by UQ Health Care in partnership with Health and Wellbeing Queensland, along with key stakeholders: The University of Queensland, Griffith University, Metro South Hospital and Health Service, and Brisbane South Primary Health Network. The clinic provides a range of group and individual allied health services, including physiotherapy, exercise physiology, dietetics, social work, and health psychology. Students from across these disciplines, and others including public health and pharmacy, are integrated into the service delivery, supervised by interprofessional clinical educators.

### SSBC Australia Coaches

#### Overview

SSBC Australia coaches will be recruited at each of the sites using recruitment strategies identified in partnership with the delivery site. For The Y, it is expected that most coaches will be existing staff identified by the site leads as suitable for program delivery. For Logan Healthy Living, it is expected that most coaches will be drawn from students undertaking their placement at the site during the study period. Importantly, SSBC Australia coaches are not required to have any professional qualifications and can include lay volunteers and students. All coaches are required to be 18 years or older, meet the site-level requirements for volunteers at their respective sites, and pass the competency assessment for SSBC Australia intervention delivery. All coaches are also required to sign a nondisclosure agreement prior to undertaking the SSBC training as part of the Canadian-based requirements.

For Logan Healthy Living, all students, from any degree, undergoing at least a 5-week work integrated learning placement during the study period (and beyond depending on findings from the study) will be required to undertake the SSBC Australia training as part of their placement experience. Students can then opt in to provide data collected as part of this standard process to the research project and to deliver the SSBC Australia program to clients. Only those who provide consent for the research will be eligible to deliver the program to clients, providing they meet other eligibility requirements.

#### Target Sample Size—Coaches

There is no target sample size for coaches. Each Y site is required to have at least 2 trained coaches, while the Logan Healthy Living site is expected to have a small number of trained clinicians and a larger number of trained student coaches (anticipated total n≈50, mostly from Logan Healthy Living).

### Program Participants (Clients)

#### Overview

Program participants (clients) will be recruited at each of the sites using recruitment strategies identified in partnership with the delivery site. Recruitment strategies are expected to include but are not limited to listserv mailouts, on-site promotion, establishment of primary care and public health care referral pathways, promotion through local community groups, and health information showcases. Referral sources and referral strategies will be tracked by the project team.

The recruitment materials will direct potentially interested clients to complete an online expression of interest (EOI) and screening survey (REDCap). This survey, which was based on the Canadian SSBC eligibility survey, is anonymous until participants reach the consent stage, with the survey stopped if a participant does not meet the inclusion criteria at each of the staged steps. Ethics for this EOI and screening survey was obtained from The University of Queensland Low and Negligible Risk Human Research Ethics Committee (#2024/HE001280).

The EOI and screening survey will include the AUSDRISK as well as questions on glycosylated hemoglobin and fasting blood glucose level (if known). Permission to use the AUSDRISK, which is a simple yet valid and reliable risk assessment tool to predict incident type 2 diabetes within the next 5 years [36], was granted from the Australian Government Department of Health and Aged Care. It comprises a series of questions about diabetes risk factors including age, sex, personal and family history of high blood glucose, First Nations background (Aboriginal, Torres Strait Islander, Pacific Islander, and Māori), other ethnic minority background (Asian, Middle Eastern, North African, and Southern European background), if taking medication for hypertension, smoking status, diet, exercise habits, and waist measurement. A diabetes risk score between 0 and 38 will then be calculated, with scores being assigned according to risk for each factor, where a higher score indicates a higher risk of developing type 2 diabetes in the next 5 years.

#### Eligibility and Consent

Adults who are not pregnant (18 years or older) with a AUSDRISK score of 12 or over (considered high risk) or between 6 and 11 (considered intermediate risk) with at least 1 modifiable risk factor (smoking, low fruit and vegetables, low physical activity, and high waist circumference), or self-reported prediabetes or a glycosylated hemoglobin of 5.7% to 6.4% or fasting blood glucose of 5.6 to 6.9 mmol/L (if provided), able to provide informed consent, and able to attend at least 1 of the delivery sites, will be considered eligible and invited to complete the consent form. Information on comorbid conditions will also be captured at this point.

Participants who provide consent will then be automatically sent an information pack (via REDCap), tailored to their preferred site, which provides a guide to the SSBC Australia program and instructions for next steps. The site that the client indicates as their preferred site will also be notified of this potential client, along with information about their risk level. Participants will be encouraged to contact the site directly to support the enrollment process, depending on site preferences. Eligibility will be reconfirmed as part of this enrollment process. Once the client has been confirmed eligible by the site, they will be onboarded into the REDCap client database.

#### Target Sample Size—Participants

The target sample size is 500 participants enrolled (400 from the Y Queensland sites and 100 from the Logan Healthy Living site) for the 2-year researcher-led recruitment window, with no maximum sample size. This target sample size was determined based on the anticipated feasibility of recruitment. This sample size of 500 across 5 sites is sufficient to provide 80%-90% power with 5% 2-tailed significance to detect a 60 minute per week change in self-reported MVPA, which requires sample size of 394-528 based on assumptions of *r*=0.4, SD 330, 20% attrition, and intraclass correlation=0.001 (design effect=1+0.001(100–1)=1.099) informed by unpublished data from similar programs delivered in Logan and The Y sites.

### Data Collection

#### Overview

A variety of data collection methods will be used, including surveys, objective assessments, checklists, qualitative interviews, site audits, website analytics, meeting minutes, and project tracking. Data collection will take place at multiple levels including the organization, provider site, coach, and client. Both online and in-person measurements will take place. Survey data specific to the trial will be collected via Qualtrics for the data from the organization and site leads and via REDCap (version 15.0.7) for the SSBC coach information and SSBC coach training, client EOI and screening, and client data. Broader site-level data (eg, memberships) will be extracted from the organizational databases. Interviews will be audio-recorded and transcribed. Meeting minutes will be taken and reviewed; website analytics (from the 3C training platform and from the SSBC website coach forums) will be downloaded and reviewed. Further details are provided below.

#### Organizational- and Site-Level Data Collection

The organizational- and site-level leads will provide or have provided informed consent for the action-research process of data collection throughout the project (ethics approval from The University of Queensland HREC #HE001455). Data collected at both the organizational and site level will include information on contextual factors, organizational readiness, acceptability, appropriateness, feasibility, and sustainability. Data will be collected via surveys, interviews, meeting minutes, site visits, and document reviews of operating protocols to understand how the program is working within the site workflow (Table 3).

**Table 3:** Data collection time points for organization leads and site-level leads.

Data collection			Preimplementation		Postimplementation								
					≈3 months		1 year		2 years		3 years		4 years
**Survey**													
	Demographics and work characteristics^a^	✓											
	Organizational readiness for change	✓						✓				✓	
	Barriers and facilitators to implementation	✓						✓				✓	
	Program perceptions	✓		✓		✓		✓		✓		✓	
	Sustainability assessment					✓		✓		✓		✓	
Preimplementation adaptation workshop			✓										
Interviews			✓		✓		✓		✓		✓		✓
Document review of site operating protocol			✓		✓		✓		✓		✓		✓
Site visit and audit			✓		✓		✓		✓		✓		✓
Sustainability adaptation workshop									✓				✓
Meeting minutes			✓		✓		✓		✓		✓		✓
Membership conversions					✓		✓		✓		✓		✓
Access and input to community of practice					✓		✓		✓		✓		✓

^a^If organization or site-level leads change, then the new leads will complete demographics and work characteristics questions.

#### SSBC Australia Coach-Level Data Collection

Data collected from coaches will include information on their demographic and work characteristics, their experiences with the training, their perceptions of the program, and their fidelity to program delivery, with data collected via surveys, website analytics, and interviews. Data collection for general coaches will continue up to 4 years (Table 4); data collection for the student coaches will continue up to 12 months (Table 5). Data will be collected on the coach community of practice and the session checklists throughout program delivery.

**Table 4:** Data collection time points for general health coaches.

		Profile or pretraining	End training	≈2-month post	12-month post	2 years	3 years	4 years
**Surveys**								
	Demographics	✓						
	Referral pathway							
	Training and experience	✓						
	Work and study	✓			✓	✓	✓	✓
	Training motivation							
	Confidence and knowledge	✓	✓	✓	✓			
	Knowledge check		✓					
	Feedback on the training		✓					
	Perceptions of program		✓	✓	✓	✓	✓	✓
Competency assessment			✓					
3C website analytics			✓					
Interviews				✓	✓	✓	✓	✓

**Table 5:** Data collection time points for student health coaches.

			Profile or pretraining		End training		End placement^a^		1-month post placement^a^		12 months
**Surveys**											
	Demographics	✓									
	Work and study	✓									
	Student study profile	✓									
	Confidence and knowledge	✓		✓		✓		✓		✓	
	Knowledge check			✓							
	Feedback on the training			✓							
	Student placement impact					✓					
	Student training impact					✓		✓		✓	
	Perceptions of program			✓		✓		✓		✓	
Competency assessment					✓						
3C website analytics					✓						
Interview							✓				

^a^Placement duration varies from 5 to 20+ weeks depending on the program requirements.

#### Client-Level Data Collection

Data from clients will be collected via online surveys as part of the eligibility, screening, and consent process and as part of their profile, preprogram, end-of-program, and at 3, 6, and 12 months post program following consent (Table 6). In-person measurements will occur at the preprogram, end-of-program, and 12-month time points. A subsample of clients will also be invited to take part in interviews at the end-of-program and at 12 months post program.

**Table 6:** Data collection time points for clients.

Client-level measure			Profile		Preprogram		End-of-program		3 months post program		6 months post program		12 months post program
			Enrollment		≈Session 1		≈Session 6		≈Time after session 6				
**Surveys**													
	EOI^a^ and screening	✓											
	Demographics	✓											
	Work and study	✓											
	Emergency contact	✓											
	Mailing list	✓											
	Connections and access			✓								✓	
	Adult Pre-Exercise Screening System			✓									
	Movement behaviors			✓		✓		✓		✓		✓	
	Dietary behaviors			✓		✓		✓		✓		✓	
	Program behaviors			✓		✓		✓		✓		✓	
	Confidence and supports			✓		✓		✓		✓		✓	
	Health-related QoL^b^			✓		✓				✓		✓	
	Loneliness			✓		✓				✓		✓	
	Health care use			✓								✓	
	Program feedback					✓							
	Program acceptability					✓							
	Prediabetes and diabetes status					✓		✓		✓		✓	
	Involvement in fitness and community			✓		✓		✓		✓		✓	
	Adverse events					✓		✓		✓		✓	
	Referral			✓		✓		✓		✓		✓	
In-person clinical measures					✓		✓						✓
Interviews^c^							✓						✓

^a^EOI: expression of interest.

^b^QoL: quality of life.

^c^Interviews in the subset only. Only those who are eligible and consent go on to be scheduled to receive the program.

### Measures and Outcomes

The measures and outcomes are mapped to the PRISM and RE-AIM framework [40], with information ordered by context, adoption and reach, implementation, effectiveness, and maintenance.

#### Context

Context will be captured under the 4 PRISM dimensions: characteristics of the implementation settings or delivery providers, perspectives on the intervention, the external environment, and implementation and sustainability infrastructure.

#### Characteristics of Implementation Settings and Delivery Providers

Characteristics captured will include the location of the delivery site, which will then be mapped to the Modified Monash Model [59] and Index of Relative Socio-Economic Advantage and Disadvantage [60] to understand remoteness and socioeconomic status of the areas in which the program is being provided; the number and type (eg, staff qualifications) of staff; the services offered (eg, group classes and types of classes); and the client base (number of members and types of memberships). Data will be collected via the site audit and reviewed annually.

Other characteristics will be collected via a CFIR-informed audit and interview. This will include, for example, understanding of other programs delivered by the site (especially those catering to a similar client base), alignment with the priorities of the site and organization, resource availability (including facilities), and existing communication channels to their members and the community.

#### Perspectives on the Intervention

Readiness for change will be assessed via the Organizational Readiness for Implementing Change tool [61] administered to both organizational- and site-level leads at preimplementation, 3 months post implementation, and then yearly. The measure consists of 12 items, each measured on a 5-point Likert scale (1=disagree and 5=agree). The Organizational Readiness for Implementing Change has demonstrated good content adequacy and reliability [61].

The pragmatic context assessment tool will be used to understand contextual barriers to change from the perspective of site leads [62]. This questionnaire, which maps to the CFIR [37], is designed to help facilitate identification of barriers and facilitators prior to implementing change [62]. It consists of 14 statements with participants asked to indicate whether they disagree (barrier), are neutral, or agree (facilitator) with each statement, and then indicate the likely effect (weak or no effect or strong effect) of this barrier or facilitator on their ability to implement the improvement. This information can then be used to map to recommended implementation strategies [62]. The pragmatic context assessment tool will be measured at preimplementation and then at 2 and 4 years to understand contextual barriers or facilitators to sustainability. Interviews, site visits, document reviews, and meeting minutes will be used to complement the survey data.

#### External Environment

Information on a range of external factors will be collected including links with community health partners, links to other support services in the area, and relevant policies and market forces. Data will be collected via interviews and audits with site leads at preimplementation and through the adaptation workshop, then annually.

#### Implementation and Sustainability Infrastructure

Information on the support provided such as ongoing training and feedback by the dedicated implementation team via communities of practice will be collected via meeting minutes (Australia) as well as analytics data from the coach forum section of the Canadian SSBC website (Canada). Each site will have its own program operating protocol (including recruitment, enrollment, evaluation, reporting, communication, and retention protocols) with this site operating protocol developed in partnership with the site, then reviewed at approximately 2 months after implementation, then annually with protocol modifications tracked. Information regarding sustainability planning will be collected via interviews with site and organizational leads at preimplementation, then annually, as well as through the sustainability adaptation workshops at 2 and 4 years.

#### Adoption and Reach

Adoption and reach measures are reported in Table 7, with data collected via a mix of surveys, interviews, and project tracking.

**Table 7:** Adoption and reach outcome measures.

Outcome		Measure	Instrument
**Adoption (organization, site, and coach)**			
	Reasons for taking up program	Organizational reasons Site reasons Coach reasons^a^	Preimplementation interview Preimplementation interview Coach profile
	Uptake of health coaches	Number of coaches interested (n) Number of coaches eligible or ineligible (n) Number of coaches SSBC^b^ Australia certified (n)	Coach consent Project tracking Competency check
	Referral pathways coach^a^	How they heard about the program	Coach profile
	Characteristics of coaches	Demographic characteristics Work and experience characteristics Study profile^c^	Coach demographics Coach work and experience Student coach study profile
	Withdrawals or reasons for withdrawals	Number of withdrawals (n: site and coach) Reasons for withdrawal site Reason for withdrawal coach	Project tracking Withdrawal survey or interview Withdrawal survey or interview
	Adverse events	External factors impacting on adoption (site renewal, natural disasters, and person-made disasters)	Project tracking
**Reach (participants)**			
	Uptake of participants	Number interested (n) Number eligible or ineligible (n) Number consented (n) Number enrolled (n)	EOI^d^ and screening EOI and screening Participant consent Number onboarded to REDCap^e^ client database
	Referral pathways	How heard about the program	EOI and screening
	Characteristics of enrolled participants	AUSDRISK^f^ score, site, comorbid health conditions Demographic, work, and study	EOI and screening Demographics, suitability for exercise, work, and study
	Characteristics of ineligible participants	Demographics, AUSDRISK score (if available)	EOI and screening
	Withdrawals	Number of withdrawals (n) Reasons for withdrawals	Participant tracking Withdrawal survey

^a^Not asked for the student coaches at Logan Healthy Living, as undertaking the training is a requirement of their placement.

^b^SSBC: Small Steps for Big Changes.

^c^Asked of students only.

^d^EOI: expression of interest.

^e^REDCap: Research Electronic Data Capture.

^f^AUSDRISK: Australian Type 2 Diabetes Risk Assessment Tool.

#### Demographic Characteristics (All Participants)

Characteristics to be measured on all participants (organizational lead, site leads, health coaches, and clients) include age (year of birth), sex assigned at birth, gender, location (provider site), ethnic origins, country of birth (Australia or other), main language spoken at home (English or other), disability status, highest education level, home postcode, and paid employment and study status.

#### Work History and Experience (Health Coaches)

All health coaches will additionally be asked about their qualifications, time worked in the health or health promotion field, completion of any courses in behavior or behavior change, amount of training in health or health promotion, cultural safety, MI (5-point scale from 1=none to 5=extensive), reasons for doing this training, and experience in using MI and working with clients with type 2 diabetes or prediabetes (5-point scale from 1=none to 5=extensive).

#### Student Study Profile (Student Health Coaches)

Student health coaches will be asked additional questions to the general health coaches that are specific to their university training, namely, student status (domestic or international), university, program of study, degree level (undergraduate or postgraduate), year of study, and number of full-time placements completed (0 to 5+).

#### Suitability for Exercise (Clients)

Clients will be asked to complete the Adult Pre-Exercise Screening System [63] before the intervention. None of the survey responses are automatic disqualifications for participation. Responses will be used by coaches to discuss any relevant safety concerns about participation with participants at their first session, including escalating to a review by an appropriate allied health professional or medical practitioner as required, based on the site-level protocols. This questionnaire also provides open text for the client to record anything else they would like to talk about with their coach or like their coach to be aware of.

#### Contextual Measures (Clients)

Clients will be asked about their social connections and access to healthy food, transport, recreation spaces, assistance with legal matters, housing or accommodation, and health services using the 6-item access subscale and the single item on connections outside of family (on a 9-point scale from 0=not at all connected or poor access to 8=very connected or easy access) from the Steps to Better Health Questionnaire [64]. Clients will also be asked about their involvement in activities outside the home (social, exercise groups, art and craft, and performing arts), their membership with any fitness facilities (the study site and other), and their referral of others to the SSBC Australia program and to the site.

#### Other

All coaches are asked to indicate their level of motivation to do the required training to become a SSBC Australia coach (0=not at all to 10=extremely). Further, health coaches who are not students will be asked their reasons for becoming a coach (open text) and how they heard about the program. This will not be asked of the student coaches, as they will all do the training as part of their placement requirements. Referral pathways will also be captured as part of the screening and consent process for clients.

#### Implementation

The implementation outcomes will consider both the SSBC Australia coach training and program delivery. SSBC Australia will be considered practicable on the APEASE criteria if it is able to be appropriately implemented (high fidelity) for both these elements.

#### Implementation—Health Coach Training

Fidelity of the training process will be considered in terms of the number of core training components completed: the 7 online Canadian modules (assessed via website analytics), the 4 core Australian-specific modules and review of the client workbook (assessed via self-reported completion), the knowledge check quiz, and the competency assessments. All components are expected to be completed for training fidelity.

Acceptability, appropriateness, and feasibility of the training will be measured following completion of the competency check process via a bespoke survey. In total, 5 items ask about the acceptability, usefulness, appropriateness, feasibility, and value (to current and future career) with responses on a 5-point Likert scale (1=not at all to 5=extremely), and 2 items will be asked on satisfaction regarding the length and content of the training in general (1=extremely dissatisfied to 5=extremely satisfied). For each of the training components the coach indicated they did, they will be asked how useful the component was (1=not at all useful to 5=extremely useful). One question will be open text for any other feedback. The self-reported time spent on each of the training components will be captured, along with details about training for their competency checks (since the sites have flexibility in how these are conducted).

#### Implementation—Program

### Provider Level (Coach, Site Lead, and Organization Lead)

Acceptability, appropriateness, and feasibility of the program from the perspective of coaches, site leads, and organization leads will be measured by the Acceptability of Intervention Measure, the Intervention Appropriateness Measure, and the Feasibility of Intervention Measure [65]. These measures, which can be used separately or together, have demonstrated high test-retest reliability (*R*=0.73 to 0.88) and good structural validity [65]. All measures use the same 5-point Likert scale (1=completely disagree and 5=completely agree), and each measure has 4 items. Scores for the items within each domain are averaged, with higher scores indicating higher acceptability or appropriateness or feasibility. Data will be collected at preimplementation, approximately 2-3 months after implementation (to capture short-term impressions), and then yearly until year 4. Student coaches will complete the measures at preimplementation, end-of-placement, and 1 month and 12 months after implementation. These factors will also be explored via interview and open-text questions approximately 2-3 months after training, then yearly until year 4 for the general coaches and site and organizational leads and at the end-of-placement for the student coaches.

Fidelity of program delivery (site level) will be considered in terms of adherence to the site operating protocols, assessed during quarterly project site meetings.

Fidelity of coaching delivery (coaches) will be considered in terms of adherence to the SSBC Australia coaching session checklist. This checklist, which is embedded into each of the 6 coaching sessions, will capture whether the core education components for each session were covered.

Quality of the coaching (coaches) will be self-rated by the health coaches at the end of each session via a checklist. Items include client engagement with the session (single item, 1=none to 6=excellent) and overall quality of the sessions as defined by the fidelity checklist (single item, 1=none to 6=excellent). A random sample of sessions will be recorded (with permission from the client), and coaches will be asked to rate their use of MI skills (5 items, all 1=strongly disagree to 5=strongly agree). These sessions will be reviewed by the research team against these same criteria (engagement, quality, and the use of MI skills). These recordings will also be used to confirm fidelity indicators.

The number and type of adaptations to the coach training and to program delivery made prior to and then during delivery will be tracked via the research team. The number and type of adaptations made in order to sustain SSBC in the Australian context (should sites decide to continue delivery) will be recorded as part of the sustainability adaptation workshop.

Sustainability of program delivery (site level) will be determined by the decision of the delivery site to continue delivery of the program at 2 years (end of research-funded support) and 4 years (end of evaluation support). Reasons for the decision will be captured as part of the yearly interviews with site and organization leads.

Costs will be considered in terms of time for coach training, program delivery and evaluation, site lead time, administration time, and provision of the in-kind membership. Coach training time will be asked of coaches as part of their posttraining survey suite. Each of the 6 client sessions will be time-stamped (start and finish) to track program delivery and evaluation time. Site lead time will be determined from their scheduled time allocated to the project (minus any training or program delivery time). Estimation of administration time will be asked at the regular site meetings and recorded by the research team.

### Client Level

Program acceptability from a client perspective will be asked via survey at the end of the program using the Acceptability of Intervention Measure and Intervention Appropriateness Measure [65]. Clients will also be asked 4 open-ended questions on barriers experienced as part of the program, how they think the program can improve, what they liked about the program, and any other feedback relating to their experience. A subset of participants, randomly selected, will be asked to take part in an interview to gain further insights on their experience, with interviews conducted at the end of the program and at 12 months (anticipated n=10-15 at each time point).

Program satisfaction will be measured at the end of the program via a survey developed specifically for this evaluation. In total, 9 questions will ask about satisfaction in terms of their overall experience, their coach, the nutrition content, the physical activity content, the program workbook, the location of sessions, the length of sessions, the length of program, and their experience with administration (scheduling), with response options on a 7-point Likert scale (1=extremely dissatisfied and 7=extremely satisfied). Clients will also be asked to indicate on a scale of 0=not likely at all to 10=definitely their likelihood of promoting the SSBC Australia program to family, friends, or colleagues.

Fidelity of program receipt will be considered in terms of the extent to which the program was received by the client as intended. It will be measured by the number of coaching and exercise sessions successfully completed by the client (a maximum 12: 6 coaching and 6 exercise) and will be tracked by the health coach as part of their session checklist.

#### Effectiveness

##### Training Effectiveness (Coaches)

Effectiveness of the training for SSBC Australia coaches will be assessed via attainment of competency and their change from pre- to posttraining in self-rated knowledge and confidence scores (1-7). These scores incorporate knowledge and confidence in cultural safety, prevention and management of type 2 diabetes and prediabetes, MI, physical activity, and nutrition (all collected via 7-point Likert scales from 1=strongly disagree to 7=strongly agree).

##### Program Effectiveness (Clients)

###### Overview

Client-level effectiveness outcomes are listed below. The primary effectiveness outcome is self-reported moderate to vigorous physical activity time. Effectiveness outcomes are collected either via survey or by in-person measurement at preprogram, end-of-program (primary end point), and 12 months after the program ends. The self-report outcomes are also collected by surveys at 3 and 6 months after the program ends, except that health-related quality of life and loneliness are skipped at 3 months (Table 6).

###### Clinical Indicators (Objective Physical Assessments)

In-person measures of body composition (weight [kg] and waist circumference [cm]), cardiorespiratory fitness (resting blood pressure and 2-minute step test), and strength and balance (hand grip strength and balance test) will be conducted at baseline, end-of-program, and 12 months with the primary time point change before program to end-of-program.

Height (cm) will be measured at baseline only (Seca 217 Stadiometer) to enable calculation of body mass index. Weight will be measured by digital scales (ADE Digital Scales with a capacity of 250 kg) with outer clothing (ie, jackets) and shoes removed. Waist circumference will be measured by a tape measure at the abdomen at its narrowest point between the lower costal (10th rib) border and the top of the iliac crest, perpendicular to the long axis of the trunk.

The Queens College protocol will be used for the 2-minute step test [66]. The 2-minute step test has been shown to have high intraclass reliability (*R*=0.9) and criterion validity (*r*=0.74) [67]. Blood pressure will be measured by an automatic blood pressure monitor (model HEM-907; Omron) after the client has been seated for at least 5 minutes. The average of 2 readings will be used.

Hand grip strength will be measured with a dynamometer (Camry model EH101), with the average of 3 trials on each hand will be used. A systematic review reported this test to be a valid and reliable measure [68]. The 4-stage balance test with ceiling [69] will be used for assessment of balance, with participants asked to hold each position for at least 30 seconds if they can. This test has been shown to have acceptable reliability (test-retest *r*=0.66), validity, and discriminant ability [70].

###### Self-Report Measures

MVPA will be captured with the Physical Activity Vital Sign (PAVS) questionnaire [71]. The PAVS is recommended by the American College of Sports Medicine as part of their Exercise is Medicine resources and has demonstrated good concurrent validity [72]. An additional question will be added to the PAVS to identify how much of that time is spent specifically in vigorous (strenuous) activity.

Strength training will be captured through the question from the PAVS on strength training [71].

Movement behaviors across the 24-hour day will be measured using a bespoke single-item (4-part) question that has shown good validity against an activPAL criterion in in-house testing and good acceptability from participants. Here, participants will be asked their time (in hours and minutes) spent in the following activities for a typical day, with the total needing to add up to 24 hours: sleeping, moderate or strenuous (vigorous) activity (noting this is prefilled from their responses to the PAVS), other lighter moving or standing, and sitting or lying down (sedentary).

The percentage of prolonged sedentary time (sitting or lying for 30 minutes or more continuously) will be captured using a single-item question shown to have adequate measurement properties in terms of both test-retest and criterion validity [73].

Sleep quality will be measured by the single-item 11-point sleep quality scale (0=terrible and 10=excellent), which has demonstrated good criterion validity (*r*=–0.76 to –0.92) and test-retest reliability (intraclass correlation=0.62) [74].

Dietary behaviors will be measured by scores from the 9-item Mini-Eating Assessment Tool, which has shown good correlation with the Healthy Eating Index assessed by a validated food frequency questionnaire (*r*=0.71) [75]. Data are collected on the frequency of consumption of fruits, vegetables, legumes, nuts and seeds, fish or seafood, whole grains, refined grains, low-fat dairy, high-fat dairy and saturated fats, and sweets and sweet foods. Permission for use was granted by the Mini-Eating Assessment Tool team (March 11, 2025). An additional item on sugar-sweetened beverages was also included in the items to enable comparison to population health surveys [76].

Participants will be asked how often they do the behaviors targeted in the program, with each of the 6 questions using a 5-point Likert scale (1=never or very rarely to 5=very often or always). The questions, developed for this study, ask about tracking and setting goals for physical activity, reading food labels, adding sugar (item reversed scored), choosing low glycemic index options, and choosing a well-balanced plate.

Psychosocial measures include those related to social support, self-efficacy, and loneliness. Social support for physical activity and for diet will be measured by 2 items adapted from the Social Support and Exercise Survey [77], with respondents asked to respond to the prompt “During the past month, how often have your friends, family or members of your household a) encouraged me to do physical activity; and, b) supported me to eat healthy foods.” Response options will be on a 5-point scale (1=none of the time to 5=very often or always).

Self-efficacy for healthy eating will be measured by the 7-item healthy eating scale of the Healthy Eating and Weight Self-Efficacy scale [78]. Response options are on a 5-point Likert scale (1=strongly disagree to 5=strongly agree). This subscale has good internal consistency (α=.81) and adequate test-retest reliability (*r*=0.72) [78]. An additional question, specific to the SSBC Australia program, was added “based on my knowledge of glycaemic index (GI), I am able to choose foods that are low GI.”

Self-efficacy for physical activity will be measured by an adapted version of the exercise self-efficacy scale, which has shown good internal consistency (α=.92) and construct and criterion validity [79]. Participants will be asked “How confident are you right now that you could achieve your physical activity goals if ...” for 4 of the scenarios (felt pain when exercising, did not enjoy it, too busy with other activities, and felt tired) and 1 scenario (were in a bad mood) adapted from the feeling stressed or feeling depressed items. Response options are on an 11-point scale from 0=not confident to 10=very confident.

Loneliness will be measured using a single-item measure, which asks “How often do you feel lonely?” 1=often or always to 5=never [80].

###### Health-Related Measures

Health-related quality of life will be measured by the EQ-5D-5L [81], with registration for the use of this project obtained on July 2, 2024. This is collected as an effectiveness outcome and for its potential use for economic evaluation. The EuroQol-5D-5L asks participants to rate their health today on a visual analogue scale, where 0 is the worst health you can imagine, and 100 is the best health you imagine. It also asks participants to rate their level of difficulty (1=no problems to 5=extreme problems) with 5 dimensions (mobility, self-care, usual activities, pain or discomfort, and anxiety or depression). Their health states reported on these items will be converted to utility scores (where 0 represents dead and 1 represents full health), using value sets [82] consistent with reported norms for the Australian population [83]. The utility scores can be used to derive quality-adjusted life years.

Diabetes and prediabetes status are not effectiveness outcomes of such a short intervention but will be assessed at each time point by a single multiresponse question, along with the date of any diagnosis that occurred.

Health care use is collected in the preprogram and 12-month surveys for its potential in estimating economic benefit. Participants are asked to report over the last 12 months their number of emergency department presentations, urgent care clinic presentations, day-only hospital admissions, overnight hospital admissions (including the number of nights spent in hospital), as well as the number of times they visited each of the following: general practitioner or family doctor, allied health professional, and specialist. The questions were designed to be broadly consistent with most health care use measures [84], adding in urgent care clinics, which are a relatively new health care service in Queensland, Australia, that are designed to reduce hospital admissions and emergency department presentations.

Adverse events will be collected for trial monitoring and to inform the APEASE criteria of side effects or safety. The coach will record any adverse events occurring during sessions. Clients will be shown anything their coach recorded and asked if they have experienced any new problems with their health since they started the program (at the end of the program) or since they were last asked (at 3, 6, and 12 months post program). They will be prompted to include any new diagnoses of medical conditions or illnesses, any periods of hospitalization or surgery, any muscle injuries, bone or joint problems, or any new symptoms or worsening of preexisting conditions and told they do not need to mention anything their coach already recorded. For up to 5 problems per time point, they will be asked to describe the problem, rate the likelihood it was related to program participation (highly unlikely or unlikely or about 50:50 chance or likely or highly likely or I do not know), and the severity of the problem (mild or moderate or severe).

#### Maintenance

Sustainability of the program will be measured yearly by the Short Program Sustainability Assessment Tool (version 2.0) [85], with the measure completed by site and organizational leads. This tool measures sustainability across 8 domains: environmental support, funding stability, partnerships, organizational capacity, program evaluation, program adaptation, communications, and strategic planning. Each domain has 3 statements (24 in total) with a 7-point response scale for each statement (1=to little or no extent to 7=to a very great extent) as well as a “not able to answer” option. The average score in each domain and the average total score will be considered.

Data on membership conversions and retention rates will be collected at quarterly intervals from the site-level client-management system and considered at both a site and organizational level.

Maintenance of client-level outcomes will be assessed in 2 ways. Primarily, effects will be considered maintained if there is still an improvement over preprogram levels that is present following a period of noncontact after completion of the intervention. Secondarily, the degree of change from the end of the program will be evaluated against a margin of error (δ) to determine whether outcomes are worsened (by an amount more than δ), further improved (by an amount more than δ), or maintained (unchanged to within ±δ). The primary end point for maintenance is 12 months, with other time points (3 and 6 months) collected to inform how long effects might be maintained.

#### Data Management

REDCap (version 15.0.7) will be used to collect all survey data for the coaches and clients, the client clinical assessment measures, and the implementation checklists. REDCap is a mature, secure web application for building and managing online surveys and databases. It provides automated export procedures for seamless data downloads to Microsoft Excel and common statistical packages and reporting features to facilitate project management. Qualtrics will be used for the organization- and site-level survey data collection. Any additional data collection (eg, interview recordings, meeting minutes, and participant tracking) will be stored on The University of Queensland Research Data Manager with access only provided to those named on the ethics. This research data manager will also be used to store any combined deidentified datasets. These deidentified datasets will be used for data pooling with the Canadian implementation of the SSBC program as required to address additional research questions beyond just the Australian evaluation. All staff and students involved in this study will be provided with sufficient training in the collection of data and entry into all applications. To comply with the Australian Code for the Responsible Conduct of Research [86], research data will be retained for a minimum period of 15 years from the date of publication. A deidentified datasets will be uploaded to a data repository following publication of the primary papers.

#### Data Analysis

Context, adoption, reach, implementation, and sustainability outcomes will be described. To evaluate client-level effectiveness and maintenance, changes from pre- to postintervention, as well as over the relevant maintenance time points, will be assessed using linear mixed models, correcting for repeated measures and cluster (site) using random intercepts, and adjusting for baseline values of the outcome. Analyses will be of evaluable cases, adjusting for predictors of missing data, with sensitivity analysis involving multiple imputation to test the robustness of conclusions to missing data handling choices. Effectiveness of the training will be assessed by describing competency attainment and testing changes from pre- to posttraining in confidence and knowledge scores, using 2-tailed paired *t* tests or nonparametric paired tests as appropriate. These tests ignore clustering due to the expected very small sample within each of the Y sites as well as the fact the training is provided centrally (unlike the intervention). Instead, exploratory analyses will test effectiveness in relevant subgroups (eg, Y coaches and student coaches from Logan Healthy Living).

## Results

The project is funded for 5 years. Ethics has been obtained, the trial has been registered, and the 5 sites have been recruited, with SSBC Australia coach training commenced at all 5 sites. Client recruitment started in September 2025 with a staggered start across sites. Results will be reported in conference abstracts and publications (with authorship determined according to scientific authorship guidelines) and annual stakeholder reports. Findings will be shared with stakeholders using a range of channels including newsletters, the community-of-practice, the Canadian website, and social media.

## Discussion

### Principal Findings

This trial will provide key data to inform whether the Canadian-developed SSBC diabetes prevention program can be implemented and whether it is effective within an Australian context. It is expected that the SSBC Australia intervention will demonstrate improvements in clients’ health behaviors, psychosocial indicators, and selected clinical outcomes while being feasible to implement in a range of community-based settings by trained non–health professionals. Findings will directly inform the decision-making of participating sites regarding ongoing delivery beyond the trial period and provide insights into potential scale-up nationally.

### Comparison to Prior Work and Contribution to the Evidence Base

Current diabetes prevention programs in Australia typically require health professionals to deliver the program [30,31], which may limit scalability due to workforce and cost constraints. If this trial of SSBC Australia is successful, it will demonstrate that an evidence-based, community-delivered program led by trained non–health professionals can be implemented in a new health care and cultural context. This aligns with calls to action to reconsider how we approach diabetes prevention [9] and extends international evidence on the type and importance of contextual adaptations to enable successful and sustained implementation [87]. Furthermore, while undergraduate and graduate students have been involved as coaches in the Canadian SSBC delivery [11,88], there has not yet been detailed exploration regarding the role of the coach training in a student’s learning experience from multiple perspectives (eg, student, clinical educator, and university), the impact of the training for their future careers, and any indirect impacts of the training and delivery on student experience (eg, on interprofessional development). Student health professionals are typically required to complete placements as part of their training, with supporting client behavior change, one of the key skills that can be developed within this setting [89]. Development of the SSBC Australia student-led model involved working with clinical educators, university placement coordinators, and students to adapt the Canadian SSBC coach training materials to be suitable for students as part of their placement experience. The coach training also builds on several of the core competencies identified that all health professionals should know for movement behavior change [90]. Importantly, students receive the benefit of learning, and potentially applying, MI skills within their clinical or project placement. This person-centered and goal-oriented communication style is used, and has shown effectiveness, across multiple fields [91]; however, it is perceived that students do not receive sufficient training in this skill [92]. This trial will help evaluate how useful students find this MI training in their future placement and roles.

### Strengths and Limitations

There are several components designed to enhance the sustainability of SSBC Australia. This includes investment in the support infrastructure within each site through upskilling staff in both the program delivery and research methods. Further, the multiphase process to adapt, integrate, evaluate, and potentially sustain the SSBC Australia program is a collaborative and shared decision-making process between the research team and the organization and site leads. This pragmatic and user-centered approach helps to ensure program suitability within existing workflows; that is, SSBC Australia is designed to not just be an “add-on” research project. The evaluation is guided by the PRISM and RE-AIM implementation framework [40], with data collection embedded into delivery and usual practice where possible to facilitate ongoing evaluation beyond the research project. The APEASE criteria [41] will be used to inform the business case for further investment and expansion to other sites and organizations. Limitations include the self-report of the primary effectiveness outcomes and the self-report of diabetes status. Further funding will be sought to collect data from device-based measures in a subsample. The nonrandomized, single-arm design limits causal inference regarding effectiveness; however, this design is appropriate for the study’s primary focus on implementation feasibility and contextual adaptation.

### Future Directions and Dissemination

Findings from SSBC Australia will inform potential expansion to additional community sites and health service contexts across Australia. Data will also be used as part of a larger pooled dataset with Canadian partners to examine cross-country variations in program implementation and implementation strategies. Dissemination will include peer-reviewed publications, conference presentations, community presentations, and plain-language summaries. Findings will also be communicated via the SSBC Canada and organizational websites, newsletters, and social media channels. If the trial demonstrates positive outcomes, it could provide a scalable model for diabetes prevention that complements existing services and leverages the emerging health workforce.

## Appendix

Multimedia Appendix 1 SPIRIT outcomes and StaRi checklist.

Multimedia Appendix 2 Peer review comments from Healthy Cities Implementation Science (HCIS) Team Grants Review Committee, Canadian Institutes of Health Research (CIHR).

## Abbreviations

APEASE: Affordability, Practicality, Effectiveness and Cost-Effectiveness, Acceptability, Side-Effects/Safety, and Equity
AUSDRISK: Australian Type 2 Diabetes Risk Assessment Tool
CFIR: Consolidated Framework for Implementation Research
EOI: expression of interest
MI: motivational interviewing
MVPA: moderate- to vigorous-intensity physical activity
PAVS: Physical Activity Vital Sign
PRISM: Practical, Robust, Implementation Sustainability Model
RE-AIM: Reach, Effectiveness, Adoption, Implementation, Maintenance
REDCap: Research Electronic Data Capture
SPIRIT: Standard Protocol Items: Recommendations for Interventional Trials
SSBC: Small Steps for Big Changes
SSBC Australia: SSBC in Australia
StaRI: Standards for Reporting Implementation Studies

## Data Availability

Data sharing is not applicable to this paper, as no datasets were generated or analyzed during this study.
