# Supplementary material for: A Context-Adapted Diabetes Prevention Program (Small Steps for Big Changes) in Australia: Protocol for a Hybrid Implementation-Effectiveness Study
Source: JMIR Res Protoc. 2026 Jan 19;15:e81195. doi: 10.2196/81195 (PMC12865356; doi:10.2196/81195)
Supplement: Multimedia Appendix 2 [file resprot_v15i1e81195_app2.pdf]

Review Type/Type d'évaluation:	SO Notes /Notes de l'agent scientifique
Name of Applicant/Nom du chercheur:	Jung, Mary Elizabeth
Application No./Numéro de demande:	473598
Agency/Agence:	CIHR/IRSC
Competition/Concours:	2021-10-13 Team Grant: Healthy Cities Implementation Science (HCIS) Team Grants - Letter of Intent/Subventions d'équipe: Subventions d'équipe en science de la mise en œuvre dans le domaine des villes en santé (SMOVS) - Lettre d'intention
Committee/Comité:	Team Grant : Healthy Cities Implementation Science (HCIS) Team Grants - LOI/Subvention d'équipe : Subventions d'équipe en SMO – domaine des villes en santé - LDI
Title/Titre:	Small Steps for Big Changes: Implementing an Evidence-Based Diabetes Prevention Program into Diverse Urban Communities

---

Assessment/Évaluation:

Strengths:

- focuses on an important clinical issue
- Small Steps for Big Changes is a novel intervention
- has the potential to advance implementation science in Canada
- focus on both implementation and effectiveness is very relevant
- PI has sex & gender expertise
- several letters from Australia and Canada lend support
- focus on pre-diabetes is very useful

Weaknesses:

- limited involvement of national organizations
- no explicit integration of BIPOC in the design
- insufficient EDI considerations throughout
- clearer articulation of what this intervention adds needs to be included

Budget:

- not included
